# Viral Reverse Transcriptases Show Selective High Affinity Binding to DNA-DNA Primer-Templates that Resemble the Polypurine Tract

**DOI:** 10.1371/journal.pone.0041712

**Published:** 2012-07-27

**Authors:** Gauri R. Nair, Chandravanu Dash, Stuart F. J. Le Grice, Jeffrey J. DeStefano

**Affiliations:** 1 Department of Cell Biology and Molecular Genetics, University of Maryland, College Park, Maryland, United States of America; 2 HIV Drug Resistance Program, National Cancer Institute-Frederick, Frederick, Maryland, United States of America; Centro Nacional de Microbiología - Instituto de Salud Carlos III, Spain

## Abstract

Previous results using a SELEX (Systematic Evolution of Ligands by Exponential Enrichment)-based approach that selected DNA primer-template duplexes binding with high affinity to HIV reverse transcriptase (RT) showed that primers mimicking the 3′ end, and in particular the six nt terminal G tract, of the RNA polypurine tract (PPT; HIV PPT: 5′-AAAAGAAAAGGGGGG-3′) were preferentially selected. In this report, two viral (Moloney murine leukemia virus (MuLV) and avian myeloblastosis virus (AMV)) and one retrotransposon (Ty3) RTs were used for selection. Like HIV RT, both viral RTs selected duplexes with primer strands mimicking the G tract at the PPT 3′ end (AMV PPT: 5′-AGGGAGGGGGA-3′; MuLV PPT: 5′-AGAAAAAGGGGGG-3′). In contrast, Ty3, whose PPT lacks a G tract (5′-GAGAGAGAGGAA-3′) showed no selective binding to any duplex sequences. Experiments were also conducted with DNA duplexes (termed DNA PPTs) mimicking the RNA PPT-DNA duplex of each virus and a control duplex with a random DNA sequence. Retroviral RTs bound with high affinity to all viral DNA PPT constructs, with HIV and MuLV RTs showing comparable binding to the counterpart DNA PPT duplexes and reduced affinity to the AMV DNA PPT. AMV RT showed similar behavior with a modest preference for its own DNA PPT. Ty3 RT showed no preferential binding for its own or any other DNA PPT and viral RTs bound the Ty3 DNA PPT with relatively low affinity. In contrast, binding affinity of HIV RT to duplexes containing the HIV RNA PPT was less dependent on the G tract, which is known to be pivotal for efficient extension. We hypothesize that the G tract on the RNA PPT helps shift the binding orientation of RT to the 3′ end of the PPT where extension can occur.

## Introduction

Short purine-rich segments, designated polypurine tracts (PPT), in the RNA genomes of retroviruses, are used by reverse transcriptases (RT) to initiate second (plus) strand DNA synthesis during the replication cycle [1]. The PPT primer, located adjacent to the U3 region of the genome (the HIV genome also contains a “central PPT” that may function in nuclear import or aid genome replication under some conditions [2], [3], [4], [5]) is formed during first strand DNA synthesis where the RNA genome is copied and subsequently degraded by ribonuclease H (RNase H) activity. Being resistant to RNase H, the PPT RNA remains bound to minus strand DNA. Since HIV RT can use short RNAs as primers [6], [7], [8], [9], [10], [11], resistance to degradation alone may be sufficient to specify usage of the PPT for plus strand priming. However, HIV nucleocapsid protein (NC) inhibits extension of most short RNAs produced by RNase H activity by removing them from the nascent DNA or blocking their extension, while PPT extension is not blocked [6], [7], [11]. Despite this, results suggest that like avian retroviruses, HIV also primes plus strand synthesis with non-PPT RNAs from other locations on the genome [12], [13]. The use of non-PPT RNAs for priming is not necessarily detrimental as the discontinuous plus strands can be resolved by nuclear enzymes. However, it is important that the PPT is at least one of the primers and that no RNA segments downstream of the PPT region are used unless they are subsequently removed by PPT-primed displacement synthesis. This assures that the PPT RNA sets the 5′ terminus for the upstream long terminal repeat (LTR) and provides the appropriate recognition site for integrase (IN) [1].

In addition to RNase H resistance (a property that likely results from the unique structure of the PPT-DNA hybrid [14], [15], [16], [17], [18], [19], [20], [21]), the HIV PPT is also a more efficient primer than other RNAs [6], [7]. This may in part result from proposed stronger binding to RT [7], however, the orientation of RT binding is also altered with the PPT. HIV RT shows a strong binding preference for the 5′ end of RNAs hybridized to a larger DNA template and typically cleaves them into smaller pieces [8], [22], [23], [24], [25]. Since HIV RT can extend RNA primers, it clearly can also bind at the 3′ end although results indicate this binding orientation is highly disfavored (consistent with the low efficiency of RNAs as primers). In contrast, although RT also prefers binding at the 5′ end of the PPT, the preference is less pronounced, allowing relocation to the 3′ end where extension occurs [26]. This orientation switch may be controlled by the G-rich 3′ ends of the HIV PPT (5′-AAAAGAAAAGGGGGG-3′). Experiments based on systematic evolution of ligands by exponential enrichment (SELEX) demonstrated that HIV RT had high affinity for duplex DNA primer-template sequences that resembled the PPT by having runs of 6–8 Gs at the 3′ end. These sequences were selected from “primer-template” pools containing over 10^14^ random sequences ([27], herein referred to a “primer-template SELEX”). Although the PPT primer is RNA and forms an RNA-DNA hybrid with nascent minus strand DNA, the 3′ dG tracts may mimic the structural or sequence preference imparted by Gs in the PPT. This could partially overcome the strong preference for RT to bind at the 5′ end of the RNA in an orientation that is non-productive for extension. *In vitro* analysis of PPT priming also indicates that the 3′ G tract is the most important factor in enhanced priming efficiency [8]. This tract is also important in directing RNase H cleavage to generate the correct PPT 3′ terminus [17], [18], [28].

Assuming that PPT DNA can at least partially mimic the sequence/structure of the PPT RNA, results from primer-template SELEX suggest that in addition to RNase H resistance, HIV RT and its cognate PPT may have co-evolved to promote the binding of RT in the proper orientation for extension. While the PPT is a preferred site for priming by HIV RT and lends a basis to this hypothesis, it was not known whether related retroviral RTs exhibit an equivalent sequence bias. We therefore tested retroviral RTs with related PPT sequences from Moloney murine leukemia virus (MuLV, PPT: 5′-AGAAAAAGGGGGG-3′), avian myeloblastosis virus (AMV, PPT: 5′-AGGGAGGGGGA-3′), and the non-viral enzyme from the LTR-retrotransposon Ty3, whose PPT sequence (5′-GAGAGAGAGGAA-3′) lacks homopolymeric nucleotide runs. These RTs were used in primer-template SELEX experiments to determine if they also selected for PPT-like sequences. Like HIV RT, both AMV and MuLV RTs selected sequences with G tracts at the 3′ end of the primer strand. These RTs also bound tightly to synthetic cognate DNA PPTs and non-cognate retroviral PPTs, but not to the Ty3 DNA PPT. In contrast, Ty3 RT showed no binding preference for its own DNA PPT over random sequences and SELEX experiments yielded no preferred primer-template sequences. The implications of these findings are discussed.

## Results

### Retroviral RTs have a strong affinity for their own or closely related DNA PPTs

**Figure 1:**
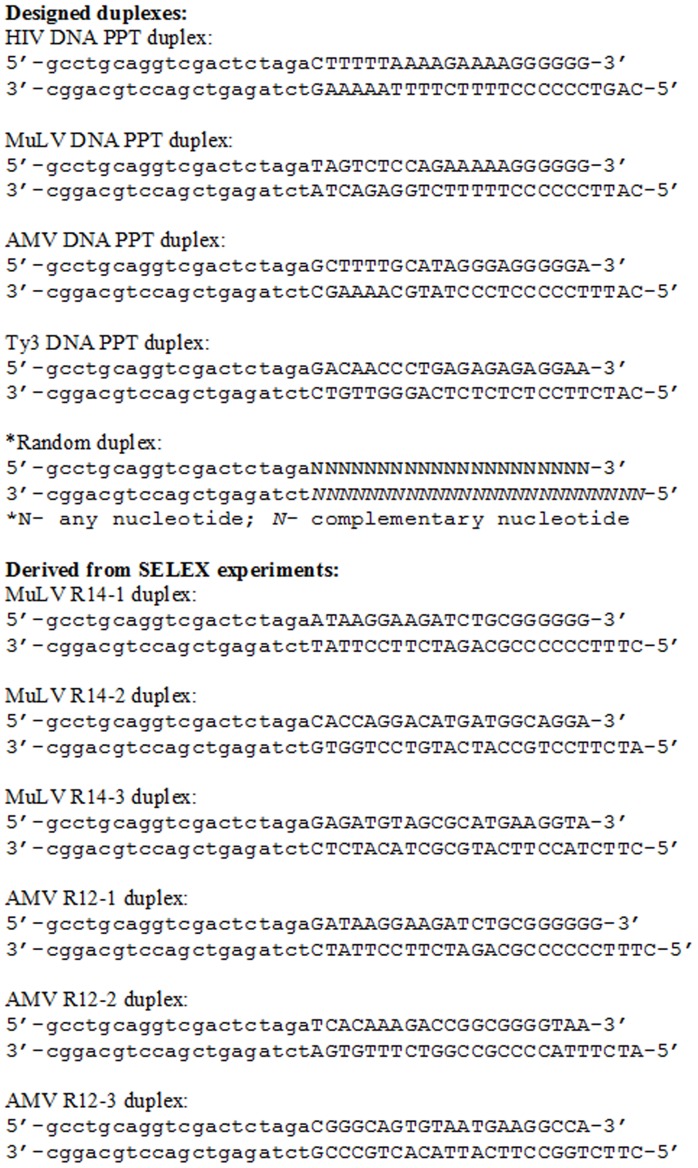
Designed and SELEX selected duplexes tested for binding to RTs. Shown is the sequence of the duplex constructs tested for binding to the various RTs (see Table 1). Each template strand was 45 nts in length while primer strands were 41 nts. All constructs had a four nt 5′ overhang. Capital letters are sequences that were derived from the PPT containing region of the genome for the particular designed duplex (other than the Random duplex) or those that were selected from the randomized region for the SELEX-derived duplexes. The sequences with small lettering are identical in all the constructs and were derived from the fixed primer region in the primer-template SELEX protocol.

**Figure 2:**
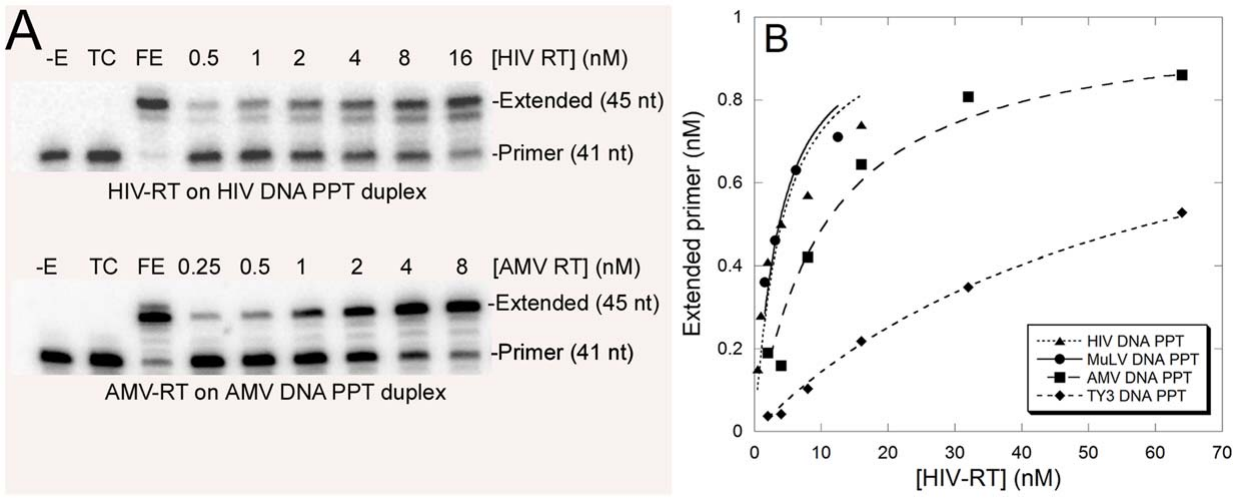
Example of assays and plots used to determine K_d_ values for the duplex shown in **Fig. 1**. (A) An autoradiogram of an experiment performed with HIV (top panel) or AMV (bottom panel) RT on their cognate designed DNA PPT constructs (HIV DNA PPT, and AMV DNA PPT duplex, respectively) (see Fig. 1). Positions for the unextended and extend primer are indicated as is the amount of enzyme used in each assay. –E, no enzyme control; TC, trap control to test for trap efficiency in which the enzyme and trap were mixed prior to addition to the reactions; FE, full extension control contained the highest amount of enzyme used in the assay incubated with the substrate in the absence of trap for 10 min. Refer to the Methods section for details. (B) A graph of extended primer *vs.* [HIV RT] for four different designed duplexes (see Fig. 1). The line shown for each duplex was made by fitting the data points to the binding equation described in the Methods section. This line was used to determine the enzyme's K_d_ for the particular construct. The data shown is from a single experiment with each construct. Experiments were repeated 2–4 times and data presented in Table 1 is an average of those experiments ± standard deviations.

In order to directly test the binding affinity of the various RTs for random duplex DNA primer-template sequences or DNA duplex sequences that mimicked the PPTs, the substrates shown in Fig. 1 were prepared. Each substrate has a 41 nt primer strand bound to a 45 nt template strand with a four base 5′ template overhang. The 21 nts at the 3′ end of the primer strand along with the 25 nts at the 5′ end of the template strand were derived from viral sequences while the remaining 20 nts of each strand were from the PCR primers used during SELEX. A four base overhang, being relatively short, may not be ideal for RT binding [29], [30]. It was used here to mimic the substrates in the primer-template SELEX experiments for which the choice of overhang length is limited by the availability of restriction enzymes that work in the selection protocol. One substrate for each viral PPT was tested along with a control containing a random sequence. Binding affinity was determined using a primer-extension assay (Fig. 2A), and dissociation constants (K_d_) were determined by curve fitting as described in Materials and Methods. A representative graph for HIV RT binding to various constructs is shown in Fig. 2B, and quantitation in Table 1. For K_d_ determinations, Ty3 RT concentrations were determined by colorimetric analysis or from the extinction coefficients [31]. For HIV, AMV and MuLV RTs the manufacturers were consulted to obtain protein concentrations. Active site titrations were not performed. For this reasons it is not possible to make definitive statements about the binding affinity of one enzyme *vs.* another to a particular substrate. However, some clear trends were evident. All retroviral RTs bound viral DNA PPT substrates more tightly than the random substrate or the Ty3 DNA PPT substrate. HIV RT showed a modest preference for its own DNA PPT substrate and the closely-related MuLV substrate over the AMV DNA PPT. AMV RT showed a similar modest preference for its own DNA PPT over those of HIV and MuLV. All retroviral RTs bound relatively poorly to the Ty3 DNA PPT but still showed some preference for this construct over a random DNA duplex. Interestingly, MuLV RT bound most tightly to the HIV DNA PPT followed by its own, then the AMV sequence. Finally, Ty3 RT showed no strong preference for any of the tested sequences, including its own DNA PPT.

**Table 1:** K_d_ values for RTs on various duplex constructs.

1Duplex	2HIV RT	MuLV RT	AMV RT	Ty3 RT
HIV DNA PPT	3.2±0.2	7.5±2.5	2.1±0.4	53±12
MuLV DNA PPT	3.5±0.5	12±2	2.6±0.2	45±4
AMV DNA PPT	8.2±2.0	17±1	1.0±0.1	58±4
TY3 DNA PPT	57±10	46±17	12±2	84±26
Random DNA	106±11	116±23	20±3	62±20
MuLV R14-1		8.1±2.0		
MuLV R14-2		77±1		
MuLV R14-3		53±17		
AMV R12-1			2.3±0.7	
AMV R12-2			3.5±0.1	
AMV R12-3			6.8±0.1	

1 Refer to Fig. 1 for duplex sequences.

2 Experiments for determining K_d_ values are illustrated in Fig. 2. All values are an average of 2–4 independent experiments ± standard deviations. Values are in nM and were calculated based on protein mass as provided by the manufacturer (HIV, MuLV, and AMV RTs) or determined as described (Ty3) [31]. Active site concentrations were not determined.

### Like HIV RT, both MuLV and AMV RTs selectively bind DNA sequences that mimic the PPT 3′ terminus, while Ty3 RT showed no preferential binding

**Figure 3:**
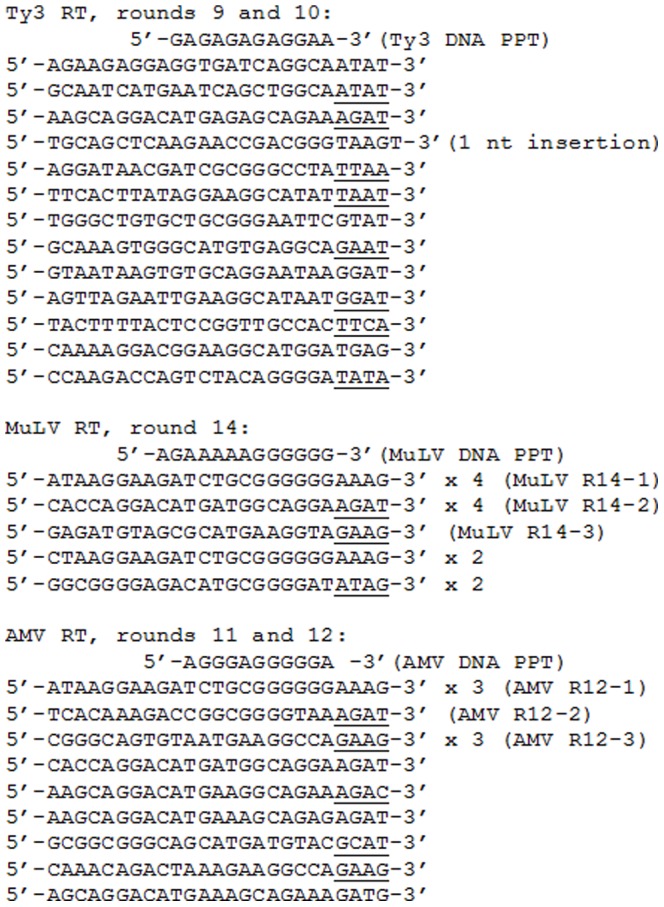
Sequences recovered from SELEX experiments. The nucleotide sequence of material recovered with the indicated enzyme in the indicated round(s) of the primer-template SELEX protocol are shown. Only the sequence in the 25 nt randomized region of the primer strand is shown. The sequence of a DNA version of the PPT for each RT is shown at the top of each set of recovered sequences for reference. During selection, the primer strand was only 21 nts long, the last four nts at the 3′ end of each sequence are underlined since they were not present on the primer during selection. However, complementary nts were present on the template strand. Specific sequences recovered with MuLV and AMV and used to prepare constructs for K_d_ determinations are designated with names corresponding to those in Fig. 1. Sequences recovered more than once are indicated by an “X” (times).

As noted earlier, a primer-template SELEX technique showed that HIV RT bound to duplex DNA primer-templates, which like the HIV PPT, contained G tracts at their 3′ primer terminus. Further characterization of the selected material showed that the G tracts were necessary and sufficient for tight binding to HIV RT [27], [32]. Even small perturbations of the run (*e.g.* changing of the terminal 3′ nt or a single internal nt change) significantly affected binding. To test whether other RTs also selected sequences mimicking the PPT, primer-template SELEX experiments were conducted for AMV, MuLV, and Ty3 RTs using a protocol similar to the one previously used for HIV RT (see Methods). Despite several rounds of selection (typically 10–15) in independent experiments, no detectable increase in binding affinity *vs.* the starting material was found with Ty3 RT. Some of the selected material from rounds 9 and 10 in one particular experiment was sequenced and is shown in Fig. 3. Since the Ty3 PPT is 12 nts long and composed of only A and G residues, the last 12 nts at the 3′ end of the sequenced material was evaluated more closely. Of the 13 sequences shown, 102/156 nts from the 3′ ends were dA (47) or dG (55). There were also several dA runs and dG runs of two or more nts, probably owing to the high purine content. However, beyond this, no strong resemblance to the Ty3 or other PPTs was noted. Overall the results suggest that Ty3 RT does not have a strong preference for any particular duplex DNA primer-template sequence, at least not in the form that was used here with a four nt 5′ template overhang (also, see Discussion).

In contrast to Ty3 RT, MuLV RT showed a clear increase in binding to material obtained from SELEX. Material from round 14 bound MuLV RT with equivalent affinity as the MuLV PPT in Table 1. Analysis of 13 clones from that round yield the sequences shown in Fig. 3, several of which were recovered more than once. Six of the 13 sequences had six consecutive dGs at the 3′ end while the remainder were also G rich within the six nts at the 3′ end (22/42 nts were dGs). The approximate K_d_ values for some of these sequences in the context of the primer-template substrates shown in Fig. 1 are listed in Table 1 while the construct is shown in Fig. 1 (MuLV R14-1, 2, and 3). Note that the selected material with the (dG)_6_ tract in the primer strand (MuLV R14-1) bound MuLV RT with essentially the same K_d_ value as the MuLV DNA PPT substrate. Although selected material lacking 3′ dG tracts (MuLV R14-2 and 3) showed lower binding affinities, they appeared to bind more tightly than the starting material. The results indicate that MuLV RT, like HIV RT shows a preference for binding to duplex DNA primer-templates mimicking the 3′ end of the RNA PPT.

Results with AMV RT were more difficult to interpret. After 5–7 rounds of selection an approximately 2-fold increase in binding affinity over the starting material was observed, but several subsequent rounds (up to round 12) yielded no improvement. Three of the 13 clones sequenced from rounds 11 and 12 had a (dG)_6_ tract at the primer 3′ terminus (all were the same sequence, denoted AMV R12-1), suggesting a clear preference for this motif. In fact, this identical sequence was also recovered from round 5 (data not shown). The affinity of AMV RT for this sequence was essentially equivalent to that for the HIV and MuLV DNA PPTs but reduced relative to its own DNA PPT (Table 1, Fig. 1). Other sequences recovered from round 12 with AMV RT also showed some preference over the starting material (AMV R12-2 and 12-3), especially AMV R12-2 for which the last seven nts at the 3′ primer end were GGGGTAA-3′. This sequence bound just slightly less tightly than AMV R12-1 and was essentially equivalent to AMV RT binding to the HIV and MuLV DNA PPTs (Table 1). Although AMV RT was less selective than RTs from HIV and MuLV in these experiments, the presence of some tight binding material with dG tracts at the 3′ end within the selected pools strongly suggests a binding preference for this motif.

### HIV RT does not show a strong preference for binding its cognate RNA PPT

The primer-template SELEX assay used duplex DNA rather than RNA-DNA hybrids to examine RT binding preferences, since technical limitations of SELEX prevent the latter substrate from being used. Results herein also showed that viral RTs bind strongly to primer-template substrates containing DNA versions of their cognate PPTs as primers (Table 1), although this probably results mostly from the dG tracts at the 3′ end (see Discussion). These results raise the possibility that retroviral RTs simply bind tightly to 3′ dG tracts while this same motif in RNA does not lead to enhanced affinity. It is also possible that since RT typically binds at the 5′ rather than 3′ end of RNA primers, the G tract may simply increase the proportion of RT binding to the 3′ end while not affecting overall binding affinity. To test this further, six primer-template sequences consisting of a 19 nt DNA strand bound to RNA or DNA primers were constructed (Table 2). Primer strands were the 15 nt HIV DNA or RNA PPT, the 12 nt Ty3 RNA PPT, a 15 nt version of the Ty3 RNA PPT with three nts added to the 5′ end, and two controls that were used in previous experiments [6]. These constructs were smaller than those used in primer extension assays constructed to mimic primer-template SELEX substrates and were comparable to RNA-DNA duplexes used in previous work [7]. Construct affinity was examined by filter binding as it is not possible to compare binding of these diverse primer-templates using primer extension assays. This is because RT does not extend RNAs, especially non-PPT RNAs, with high efficiency and extension can therefore not be used as a direct indicator of binding. This would also be a problem if RNase H cleavage were used as the assay readout since the PPT RNA is not efficiently cleaved. A further complication is that Mg^2+^, which contributes significantly to RT binding [33], must be omitted from comparative binding assays as it would stimulate cleavage of non-PPT RNA/DNA hybrids. However, this can be overcome by using an RNase H minus version of HIV RT that has an aspartate to asparagine change at position 478 in the RNase H active site (RT^E478>Q^). This well characterized enzyme has properties very similar to wild type and binds nucleic acid with comparable stability in the presence or Mg^2+^, although binding more tightly in its absence [33], [34]. Duplexes were tested in the absence of Mg^2+^ with wild type RT and in the presence of 6 mM Mg^2+^ with RT^E478>Q^. Measured K_d_ values for all 6 duplexes were much higher than values determined with the primer extension assay. This may result from the much smaller size of the substrates and differences in the experimental approach. Duplexes bound to RT^E478>Q^ more tightly than wt, probably because of the inclusion of Mg^2+^ in the reactions. The duplex containing the HIV DNA PPT primer bound more tightly than any of the RNA primed duplexes with either wild type or RT^E478>Q^ . Binding affinity to the HIV RNA PPT duplex was significantly greater than binding to the Ty3 PPT duplex and Control 2. The fold magnitude of the preference for the PPT was similar to what was previously observed by others using different sequences [7]. Interestingly, a duplex with a 15 nt version of the Ty3 PPT (Ty3+3) and Control 1, which was identical the HIV PPT at the 5′ end but did not contain a G tract at the 3′ end, bound with similar affinity compared to the HIV PPT duplex. These duplexes and the HIV RNA PPT duplex were also most affected by the presence of Mg^2+^, showing ∼7–15 fold stronger binding with RT^E478>Q^. Binding to Control 2 and the Ty3 PPT duplex was very weak without Mg^2+^ and increased only 2–3 fold in its presence. Overall, these results along with the previous report [7] indicate that the strength of HIV RT binding to RNA-DNA duplexes similar in size to the PPT is sequence-dependent. However, the current results find no evidence for strong preferential binding to the HIV PPT over other non-G tract containing RNAs. The change in overall binding affinity imparted by G tracts is clearly much more pronounced for duplex DNA.

**Table 2:** Filter binding analysis of K_d_ with HIV RT wild type and RNase H minus.

1Duplex sequence	Name	2K_d_ wt (nM)	2K_d_ E>Q (nM)
5′-AAAAGAAAAGGGGG-3′ (r or d) 3′-TTTTCTTTTCCCCCTGAC-5′ (d)	HIV DNA/RNA PPT	DNA- 132±30 RNA- 1290±70	DNA- 44±12 RNA- 180±90
5′- GAGCGCGCGGCC-3′ (r) 3′-GGACTCGCGCGCCGGCGAC-5′ (d)	Ty3 RNA PPT	2380±840	850±425
5′-CCUGAGCGCGCGGCC-3′ (r) 3′-GGACTCGCGCGCCGGCGAC-5′ (d)	Ty3+3 RNA PPT	900±205	105±49
5′-AAAAGAAAAGCGCGC-3′ (r) 3′-TTTTCTTTTCGCGCGTGAC-5′ (d)	Control-1	1760±290	120±37
5′-AAUUCGAGCUCGGTA-3′ (r) 3′-TTAAGCTCGAGCCATTGAC-5′ (d)	Control-2	2610±710	1090±170

1 Duplexes were prepared by hybridization and gel purification of the hybridized material as described in the Methods section. r, RNA strand; d, DNA strand.

2 Results are an average of 2–4 independent experiments ± standard deviation. wt, wild type RT; E>Q, RNase H minus E478>Q RT. Assays with wt were without Mg^2+^ while 6 mM Mg^2+^ was present with E>Q. Values were calculated based on protein mass as provided by the manufacturer (wt) or determined as described (E>Q) [34].

## Discussion

The current results show that like HIV RT, both AMV and MuLV RTs bind tightly to DNA PPT mimics. Primer-template SELEX experiments with both enzymes indicated that the dG tracts at the 3′ end of the DNA PPTs was important for tight binding. Both enzymes selected sequences with (dG)_6_ tracts at the 3′ end, similar to the 6–8 nt tracts selected with HIV RT [27]. The remaining portion of the selected primer-templates bore little resemblance to the rest of the PPT (*i.e.* the 5′ end), suggesting that high affinity binding is driven by the dG tract. Consistent with this, sequences selected with MuLV RT bound as tightly as the MuLV DNA PPT construct despite little similarity other than the 3′ dG tract. In contrast, although AMV RT selected a sequence with a dG tract, the final selected pools contained mostly other sequences (Fig. 3). Even the single selected sequence with a 3′ (dG)_6_ tract (AMV R12-1, Fig. 1) did not bind as tightly to the enzyme as the cognate AMV DNA PPT construct (Table 1). There could be several reasons for this including biases for selection that are related to SELEX and the limited number of sequences represented in the starting pool. Although 500 pm of starting material corresponds to approximately 3×10^14^ molecules and nearly that many different sequences for a starting pool with a 25 nt random region, it is still does not represent all possible sequence diversity, which would be 4^25^ or approximately 1×10^15^. In addition, constructs with runs of several dGs at the 3′ end would in theory be present at much higher levels than specific constructs with a more defined sequence like the AMV PPT (5′-AGGGAGGGGGA-3′). The greater abundance of the constructs containing the high affinity motif would increase the possibility of selection, while rare constructs that may bind with modestly higher affinity might be harder to recover.

Although the RTs used in this work are evolutionary related and share strong amino acid identity within specific domains [35], there are also significant differences. For example, HIV and AMV RTs function as heterodimers while MuLV RT and the more distantly related Ty3 RT are monomeric [36]. The spatial separation between the polymerase and RNase H active sites also differs between Ty3 and the viral RTs, being 17–18 nts for viral RTs and approximately 21 nts for Ty3 RT [31]. LTR-retrotransposons like Ty3 are close ancestors to retroviruses and share a common replication scheme. Amino acid homology predictions suggest that their RTs are the most closely related to retroviral RTs among retrotransposons and retroelements [37], [38]. However, the RT family shows very high sequence variability such that sequence identity, even between relatively closely related members is typically not very high [39]. The retroviral RTs most closely resembling Ty3 RT are probably those of MuLV and bovine leukemia virus, with which Ty3 RT shares approximately 26% sequence identity in the polymerase domain [35]. This diversity, at least in this case, is reflected in a PPT sequence that bears little resemblance to the viral PPTs. Structural studies also indicate significant differences between the HIV and Ty3 PPTs that may allow specific contacts with Ty3 RT [19], [20], [21]. Retroviral RTs use PPTs that are very similar, especially those of HIV (5′-AAAAGAAAAGGGGGG-3′) and MuLV (5′-AGAAAAAGGGGGG-3′). HIV RT can also efficiently use the MuLV PPT to initiate plus strand DNA synthesis *in vitro* [28]. It is therefore not surprising that these enzymes bind the counterpart DNA PPTs with similar affinity (Table 1). The somewhat greater affinity of MuLV RT for the HIV DNA PPT may not be very meaningful and does not necessarily indicate that the RNA PPTs would show the same trend. The AMV PPT (see above) is clearly different in that it has G tracts of three and five nts separated by a single A residue and a 3′ terminal A. With HIV RT, changing the terminal 3′ dG on SELEX selected duplex DNA constructs weakened affinity [32] and both HIV and MuLV RTs showed reduced affinity for the AMV DNA PPT (Table 1). In contrast, AMV RT prefers its own DNA PPT, showing a modest but significant difference in binding *vs.* those of HIV and MuLV. Assuming again that DNA PPTs are reasonable mimics of RT binding to the RNA PPT, one possible reason for AMV using a more divergent PPT sequence may be a higher binding affinity for that sequence to its RT.

In contrast to retroviral RTs, Ty3 RT did not bind with higher affinity to its DNA PPT sequence (5′-GAGAGAGAGGAA-3′), or any other DNA PPT (Table 1). Primer-template SELEX experiments also did not yield any tight binding primer-template sequences. Based on this it is possible that Ty3 RT does not have a strong preference for binding a particular DNA-DNA sequence, at least not in the form of the constructs used here. It is also possible that a duplex DNA substrate cannot mimic the structural properties of the Ty3 PPT that confer high affinity binding to the 3′ terminus. For HIV, AMV, and MuLV RTs, our results suggest that this motif is the 3′ rG tract. It is easy to imagine that this simple motif could be structurally mimicked by the same DNA sequence. Although the Ty3 PPT has a unique structure that is distinct from other RNA sequences [19], [20], [21], no runs of more than two nts are present, suggesting it may form a structure that is unique to RNA. Attempts to measure the binding of Ty3 RT to RNA-DNA duplexes in the absence of Mg^2+^ using the constructs shown in Table 2 were inconclusive as the protein bound constructs with very low apparent affinity in the filter binding assay (data not shown).

Despite the strong enhancement of RT binding by G tracts on DNA-DNA duplexes, G tracts on RNA-DNA duplexes did not result in a clear binding preference for HIV RT (Table 2). HIV RT did bind its own RNA PPT duplex much more tightly than the Ty3 duplex, but the Ty3 PPT is three nts shorter than HIV. A duplex with three additional nts added to the 5′ end of the Ty3 PPT (Ty3+3 RNA PPT) bound HIV RT similar to the HIV PPT duplex despite having no G tract. This suggests that low affinity for the Ty3 duplex results mostly from it being too shorter. Two other controls yielded mixed results with one (Control 1) showing binding comparable to the HIV PPT duplex and the second (Control 2) binding much more weakly. RNA-DNA duplexes examined previously by others indicated a preference for HIV RT binding to a duplex containing the HIV PPT [7]. Binding in this case was examined by a fluorescence anisotropy based approach. Difference in the finding may have resulted from the approach used or the different sequences. Our results also indicate that some sequences (Control 2 for example), bind RT much more weakly than the PPT duplex while other do not. A larger set of sequences measured on duplexes with different types of overhangs is needed to more clearly assess binding of RT to different RNA-DNA duplex sequences.

One explanation for the G tract having a less pronounced effect on binding to RNA-DNA *vs.* DNA-DNA duplexes would be that the different orientation of binding of RT on the duplex types influences affinity. Since HIV RT is mostly bound at the 5′ end of RNA-DNA duplexes including the PPT (see Introduction), the 3′ G tract may have less of an impact on overall binding affinity. The reverse is the case for DNA-DNA where RT strongly orients toward the 3′ primer terminal end. The greater propensity for RT to flip to the 3′ end of the PPT *vs.* other RNAs primers [26] may result from the G tract transiently stabilizing this highly unfavorable mode. In this scenario overall affinity would be minimally influenced by the PPT G tract while affinity/stability in the transient 3′ binding mode could be significantly augmented. That there was no clear enhancement of affinity with the RNA PPT may also suggest that the exceptionally stronger binding to the DNA PPT and the related sequences selected from SELEX pools is unrelated to the RNA PPT. However, viral RTs selected DNA sequences with dG tracts that were nearly identical in length to the G tracts on the RNA PPT. It seems unlikely that such a specific motif would by chance invoke high affinity binding on DNA-DNA and also be present on the viral PPT which is used with high specificity for second strand priming. On the subject of binding affinity, it is also interesting that in addition to binding tightly to duplexes with 3′ dG tracts, HIV RT also binds strongly to specific nucleic acid aptamers containing G-quartet structures [40], [41], [42]. Whether the strong binding is due in part to a shared feature of these constructs is unknown.

Results presented here support the notion of strong co-evolution between retroviral PPTs and their cognate RT. This has led to a set of closely related PPT sequences consistent with the shared evolutionary lineage of retroviral RTs [37], [38], [43], [44]. Still, subtle but meaningful differences between the AMV PPT and those of MuLV and HIV appear to have resulted in PPT sequences more closely matched to that enzyme with respect to higher affinity binding to the 3′ end. Interestingly, Rous sarcoma virus (RSV), which uses the same PPT as AMV, is also able to use the MuLV and HIV PPTs to replicate, though with reduced efficiency. The lower efficiency is probably due at least in part, to non-specific cleavage of the PPT sequence [45]. On this same topic, in selection and competition experiments using MuLV clones that had PPT alterations, results indicated that several PPT sequences other than the wild type were viable [46]. There was much stronger selection for the 3′ G tract containing portion of the PPT than the A-rich 5′ end, but some sequences containing modest disruptions of the G tract were tolerated with little effect on viability and no apparent competitive disadvantage in comparison to wild type. In our SELEX experiments sequences with uninterrupted 3′ dG tracts dominated the selected pool with MuLV RT, and this also occurred with HIV RT [27]. This would suggest that some of the highly competitive sequences selected in cells may interact with RT sub-optimally, although they would likely interact more productively than “random” sequences. Perhaps in a simple cellular infection system a sub-optimal interaction may be sufficient to confer full infectivity.

In conclusion, the results presented here show that viral RTs have a marked binding affinity preference for DNA-DNA primer-template duplexes with primers possessing a 3′ G tract that mimics the PPT. We hypothesize that one of the roles of the G tract in the RNA PPT is to promote more stable binding of RT at the 3′ end of the PPT which in turn enhances the efficiency of extension. This, and other factors like resistance to NC's inhibitory effects on RNA priming, set the PPT apart from other potential RNA primers for which extension is much less efficient [6], [7].

## Methods

### Materials

Wild type HIV RT was from Worthington Biochemical Corporation. The HIV RNase H mutant (RT^E>478Q^) was prepared as previously described [34]. Ty3 RT was prepared as previously described [31]. AMV RT and dNTPs were from Roche Applied Science. Taq polymerase was from Fermentas. Restriction enzymes, Klenow polymerase, MuLV RT, Pfu polymerase, T4 polynucleotide kinase (PNK), and *E. coli* RNase H were from New England Biolabs. The Rapid DNA ligation kit was from Promega. The PCR Blunt End Cloning kit was from Agilent. The Miniprep DNA preparation kit was from Qiagen. Radiolabeled compounds were obtained from Perkin Elmer. Sephadex G-25 spin columns were from Harvard Bioscience. Nitrocellulose filter disks (25 µm, 0.45 µm pore size, Protran BA 85) were from Whatman. All oligonucleotides were from Integrated DNA Technologies. All other chemicals were from Sigma, VWR, Affymetrix (USB) or Fisher Scientific.

### Preparation of radiolabeled DNA strands

Twenty-five pmols of the 41 nt primer strand oligonucleotide was 5′-^32^P-end-labeled using T4 PNK. The labeling reaction was performed at 37°C for 30 minutes, as per the manufacturer's protocol. Reactions were shifted to 70°C for 15 minutes in order to heat inactivate the PNK. The DNA was then centrifuged on a Sephadex G-25 column in order to remove any excess radiolabeled nucleotide.

### Preparation of RNA-DNA and DNA-DNA duplexes

Duplexes were prepared by mixing two pmol of 41 nt 5′-^32^P-end-labeled DNA from above and two pmol of 45 nt template (see results section for full list of sequences) in 15 µls of buffer containing 50 mM Tris-HCl pH 8.0, 80 mM KCl, and 1 mM dithiothreitol (DTT), 0.1 mM EDTA (ethylenediaminetetraacetic acid). Reactions were placed at 80°C for 5 minutes and then allowed to slow cool to room temperature prior to use.

### Production of Random SELEX library for initial round of selection

Oligonucleotide5′-GCATGAATTCCCGAAGACGC(N)_25_ TCTAGAGTCGACCTGCAGGC-3′ (N: any nucleotide) (approximately 2000 pm) was hybridized to approximately 3,000 pm of oligonucleotide 5′-GCCTGCAGGTCGACTCTAGA-3′ that was 5′ end-labeled with ^32^P using T4 PNK as described above. Note that this oligonucleotide differs slightly from the one used as starting material for HIV RT selections in that the random nucleotide region is 25 rather than 30 nts [27]. Hybridization was performed in 50 mM Tris-HCl (pH = 8), 1 mM DTT, and 80 mM KCl in a volume of 100 µls. The mixture was heated to 80°C then slow cooled to room temperature. The material was divided into five tubes and the primer in each was extended with five units of Klenow polymerase using the following conditions: 50 mM Tris-HCl (pH 8.0), 1 mM DTT, 25 mM KCl, 6 mM MgCl_2_, and 400 µM dNTPs in a volume of 100 µls. Reactions were for one hour at 37°C. The material was then combined, extracted with phenol∶chloroform∶isoamyl alcohol (25∶24∶1 v∶v∶v), and precipitated with two volumes of ethanol and 1/10 volume 3 M Na acetate (pH 7.0). The recovered material was divided into four reaction tubes and digested with *Bbs* I (50 units) in the appropriate buffer in a volume of 150 µls overnight at 37°C. Material was run on a 12% native polyacrylamide gel along with a small amount of uncut starting material. About 50% of the material was digested to the appropriate size as judged by phosphoimaging. Digested material was recovered by eluting from crushed gel slices overnight in buffer containing 50 mM Tris-HCl (pH 8.0), 1 mM DTT, 80 mM KCl, and 0.1 mM EDTA (pH 8.0) as described [47].

### Selection of material with AMV, MuLV and Ty3 RTs via filter-binding

The basic protocol for the primer-template SELEX was described previously [27] and minor differences are detailed below. In the initial round approximately 500 pm of substrate from above was incubated with 20 pm of RTs in a buffer containing 50 mM Tris-HCl (pH 8.0), 80 mM KCl, 6 mM MgCl_2_, 1 mM DTT, and 0.1 mM EDTA. Incubation was for one hour at room temperature in a volume of 20 µls. The reaction was loaded on a nitrocellulose filter presoaked in binding buffer to minimize loss of binding and washed 2× with 25 mM Tris-HCl (pH 8.0), 10 mM KCl. Bound material was extracted by resuspending the nitrocellulose filter in 500 µls of 10 mM Tris-HCl (pH 8.0), 1 mM EDTA (pH 8.0) and subsequent phenol∶chloroform∶isoamyl alcohol (25∶24∶1 v∶v∶v) extraction. Rounds 10–14 of MuLV SELEX were performed by gel-shift and the KCl concentration was increased to 150 mM. Material was run on a 6% native polyacrylamide gel at 100 V as described [47]. For gel-shift assays a control reaction containing approximately 25 pm of material without RT was also run. Shifted material was excised and eluted as described above. The recovered material was extended with Klenow polymerase (1 U) to produce blunt ends as described above except that 100 µM dNTPs were used, the reaction volume was 50 µls, and the incubation time was 15 min. The sample was extracted and precipitated as described above, then resuspended in 10 µls of water. The recovered material was ligated using a rapid ligation kit (Promega) to 5–10 fold excess of the following two primers that were hybridized together to form a duplex: 5′-ATAGCATGAATTCGCAGAAGACCC-3′ and 5′-GGGTCTTCTGCGAATTCATGC-3′. Ligations were in a volume of 30 µls for round one and 10 µls for all subsequent rounds using the manufacturer's protocol except that reactions were for 30 min. Note that hybridization of the primers will form a duplex with a blunt end and an end with a three base 5′ overhang. This was to help direct the ligations [27]. Also, the protocol was designed to direct ligations since the only 5′ phosphorylated nucleic acid end in the reactions was on the blunt ended randomized region of the substrate (“N” containing region). A very small proportion of 5′ ends would have been phosphorylated at the other end of the selected material due to 5′ end labeling with ^32^P (see above). After ligation, PCR reactions were prepared in 400 µls total volume using the Taq polymerase buffer provided by the manufacturer. Approximately 200 pm of each primer, (5′-GCCTGCAGGTCGACTCTAGA-3′ (^32^P end-labeled) and 5′-GCATGAATTCGCAGAAGACCC-3′) were added to reactions along with the entire ligation mixture. The reactions were divided into four tubes (100 µls each) and PCR was performed at 94°C (1 min), 50°C (1 min), and 72°C (1 min) for a total of 12–14 cycles followed by an additional cycle of 5 min at 72°C. Material was combined, extracted, and ethanol-precipitated before digestion with *Bbs* I (50 units total in a 100 µls reaction as described above). Digested material was run on a 13% native polyacrylamide gel and cleaved material was recovered as described above and used in subsequent rounds of selection. A total of 10–14 rounds of selection were performed. In each round after round one, approximately 1/20 equivalent of RT (mole∶mole) was incubated with the recovered substrate for the next round of selection. Also, after round two, 20% of the recovered PCR material was saved as a source to regenerate the selected material from that round or for use in K_d_ determinations (see below).

### Determination of equilibrium dissociation constants by primer extension

Selected material (1 nM) from various rounds of selection (see Results) or other designed primer-templates (see Fig. 1) were end-labeled on the primer strand and mixed with various amounts of HIV, AMV, MuLV and Ty3 RT in 8 µls of buffer containing 50 mM Tris-HCl (pH 8.0), 1 mM DTT, 80 mM KCl, 6 mM MgCl_2_, and 0.1 mg/ml BSA for three min at room temperature. Reactions were initiated by the addition of two µls dNTPs (100 µM final in reactions) and heparin “trap” (1 mg/ml final concentration) in the same buffer as above. Reactions with AMV RT were conducted in the same manner except the trap was poly(rA)-oligo(dT)_20_ (8∶1 rA∶dT (w∶w), final concentration 0.4 µg/µl). Trap was added to sequester RT molecules not bound to the substrate and those that dissociate, limiting extension to a single binding event [48]. Samples were incubated for two min then stopped with an equal volume of 2× gel loading buffer (90% formamide, 10 mM EDTA (pH 8.0), 0.25% (w/v) each of bromophenol blue and xylene cyanol). Samples were run on a 12% denaturing polyacrylamide gel as described below, and dried gels were imaged using a Fujifilm FLA-5000 phosphoimager. The amount of bound enzyme at the various concentrations of RT was determined from the level of extended products. Controls for the effectiveness of the trap and full extension of the substrate were also performed as described in the various figures. Values for K_d_ were determined by plotting the concentration of extended product (nM) *vs.* the concentration of HIV RT and fitting the data by nonlinear least square fit to the quadratic equation: [ED] = 0.5([E]_t_+[D]_t_+K_d_)−0.5(([E]_t_+[D]_t_+K_d_)^2^−4[E]_t_[D]_t_)^1/2^, where [E]_t_ is the total enzyme concentration and [D]_t_ is the total primer-template concentration [49]. Note that this approach does not yield a direct K_d_ value as it is dependent on a secondary measurement (polymerase extension) to assess binding. This would be a concern if there were secondary binding sites on the substrates that could strongly compete with the 3′ recessed terminus for RT, or if a substantial proportion of RT interactions with the 3′ terminus were non-productive with RT dissociating before incorporating nucleotides. However, each of these concerns is of low probability for the substrates used here. Since the dissociation of the enzyme-nucleic acid substrate complexes is slow relative to the rate of polymerization [50], the amount of synthesis is proportional to the amount of bound RT at the initiation of reactions.

### Determination of equilibrium dissociation constants by filter binding

Dissociation constants were determined for the constructs shown in Table 2. The primer strand was 5′ end labeled (as described above) with ^32^P. Hybrids were prepared as described above by mixing the template strand (25 pm) with labeled primer strand (25 pm for the Ty3 PPT, TY PPT+3 and the two Control RNAs, 50 pm for the HIV DNA PPT and 200 pm for the HIV RNA PPT). A large excess of the HIV RNA PPT was used because most of this primer formed G-quartets rather than hybridizing to the template. Hybrids were fractionated on a 20% polyacrylamide non-denaturing gel in Tris-Borate-EDTA buffer as described [47]. Wet gels were exposed to an imager screen and hybridized (shifted upward on gel) material was located. Hybridization to templates was efficient for all the hybrids except for the HIV RNA PPT and to a lesser extent the HIV DNA PPT which also formed some G-quartets. Even with 2- and 10-fold excess primer, for the HIV DNA and RNA PPT, respectively, the amount of recovered hybrid was lower. The hybridized material was excised and eluted in 250 µls of 50 mM Tris-HCl (pH 8.0), 80 mM KCl, and 1 mM DTT by the crush and soak method [47]. The eluate was passed through a 0.45 µm filter and used directly for experiments after determining the amount of material by scintillation counting. The integrity of the RNA-DNA hybrids was confirmed by cleavage with *E. coli* RNase H followed by electrophoresis on 15% denaturing polyacrylamide gels (data not shown) [47]. Experiments were conducted by mixing hybrids (5 nM final concentration) with various amounts of HIV RT in 10 µls of buffer containing Tris-HCl (pH 8.0), 80 mM KCl, and 1 mM DTT. Enzyme amounts that flanked the approximate K_d_ value (estimated from initial experiments) were used. After five min at room temperature, the sample was vacuum filtered through a 25 mm nitrocellulose filter disk presoaked in reaction buffer. Filters were washed with one ml of buffer containing 25 mM Tris-HCl (pH 7.5) and 10 mM KCl at a rate of approximately 200 µl/second, air dried, and the amount of bound material was determined by liquid scintillation counting. K_d_ determinations were obtained as described above.

### Sequences analysis of products recovered from SELEX

Products selected from various rounds of SELEX were sequenced after cloning using the Stratagene Blunt End Cloning kit and transformation into *E. coli* XL-1 Gold competent cells.

Plasmid DNA for sequencing was prepared from white or pale blue bacterial colonies using the Qiagen miniprep kit.

### Gel electrophoresis

Six or 12% native polyacrylamide (29∶1 w∶w acrylamide∶bisacrylamide) or 12% denaturing polyacrylamide (19∶1 w∶w acrylamide∶bisacrylamide, 7 M urea) gels were prepared and subjected to electrophoresis using Tris-Borate-EDTA buffer as described [47].
